# Endovascular Repair of the Traumatic Dissection of the Subclavian–Axillary Artery: Report of Four Cases

**Published:** 2017-04

**Authors:** Mohammad Ali Ostovan, Javad Kojuri, Pooyan Dehghani

**Affiliations:** *Cardiovascular Research Center, Shiraz University of Medical Sciences, Shiraz, Iran.*

**Keywords:** *Vascular system injuries*, *Angioplasty*, *Stents*, *Subclavian artery*, *Axillary artery*

## Abstract

Traumatic injuries to the subclavian-axillary artery, although uncommon, are associated with high morbidity and mortality rates if not managed promptly. The emergence of endovascular techniques offers an alternative to the surgical management of these injuries. We herein present 4 cases of traumatic subclavian-axillary artery injury with limb ischemia successfully managed via the endovascular approach with non-covered self-expandable stents. We achieved excellent immediate results with limb salvage and without complications. At follow-up by duplex sonography, all the patients had good peripheral pulses and patent revascularized arteries.

## Introduction

Traumatic injuries to the subclavian–axillary artery, albeit uncommon, are allied to high morbidity and mortality rates if not managed quickly and efficiently.^1^ Surgical approaches are challenging and technically complex due to significantly traumatized surrounding structures, including brachial plexus injuries, bony fractures, and pulmonary contusions.^2^ Recently, endovascular techniques have provided a valuable alternative to surgery for the management of these injuries.^3^^-^^4^ Covered stent grafts are usually used for the endovascular repair of the subclavian traumatic injuries.^5^ However, in this case series, we report the successful use of non-covered self-expandable stents in four patients with subclavian–axillary artery injuries.

## Case Reports

***Case #1***

A 21-year-old man, a case of car accident and dislocation of the right shoulder joint, was referred to us due to cold, pulseless right upper extremity after closed shoulder joint reduction. The patient had pain at rest. In the physical examination, there was mild motor weakness and sensory loss. Digital subtraction angiography showed extensive dissection in the right subclavian–axillary artery. The patient was referred for endovascular repair.

**Figure 1:**
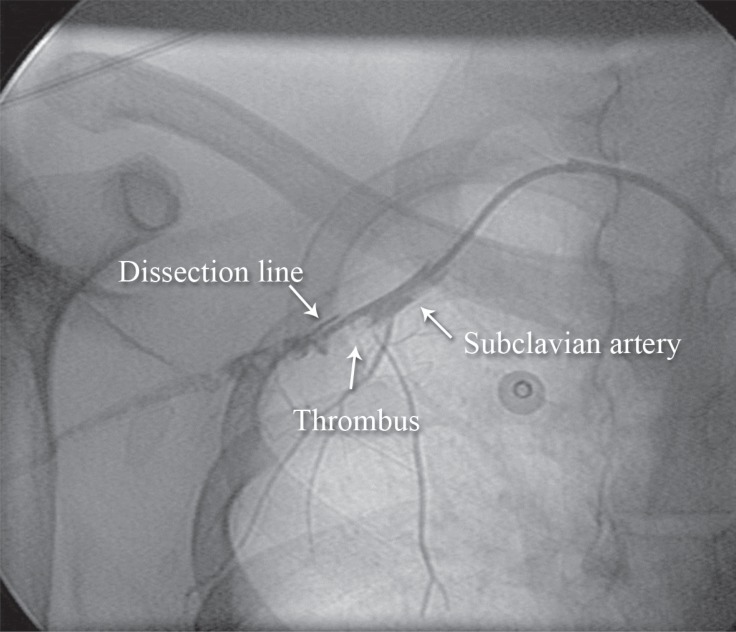
Anteroposterior view of the right upper extremity shows an extensive dissection in the right subclavian–axillary artery (Case #1).

**Figure 2:**
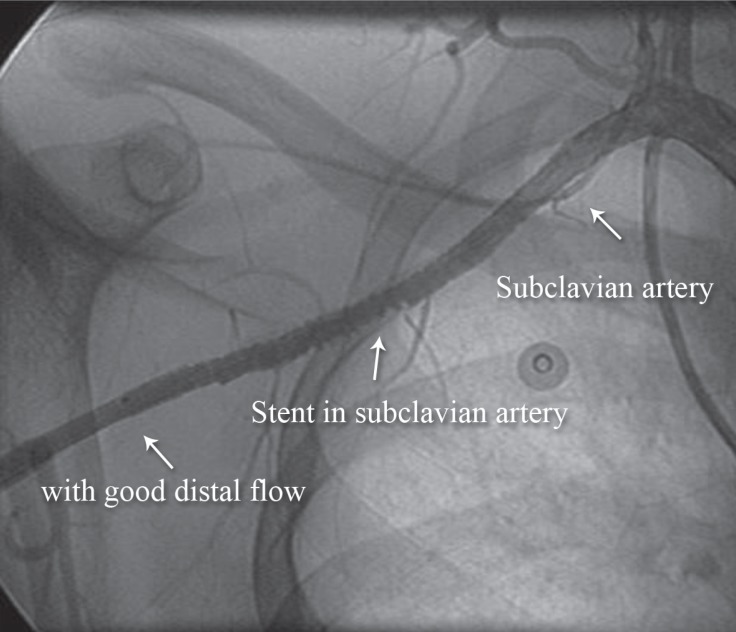
Anteroposterior view of the right upper extremity shows successful angioplasty of the right subclavian artery (Case # 1).

At the time of angioplasty, the subclavian artery was seen to be dissected and completely occluded with clot (Figure 1). It was, therefore, extremely difficult to pass a stiff Terumo wire through the lesion. We passed a Glidecath and, without predilatation, deployed an 8 × 80 mm non-covered self-expandable stent and postdilated it with a 7 × 30 mm balloon. The result was good, and there was no distal embolization or leakage (Figure 2).

The previously reported cases of the endovascular repair of the subclavian artery traumatic dissection in the literature all have been done with covered stents. To the best of our knowledge, it is the 1st time that non-covered self-expandable stents were successfully used.

***Case #2***

A 53-year-old man, a case of car accident, was referred to our center with right upper extremity trauma and signs of numbness, pain, and discoloration in the hand and forearm. There was severe arm and shoulder edema. The surgeon explored the axillary artery for repair, but severe edema and the disruption of 10 cm of the axillary artery rendered surgical repair or bypass impossible. Consequently, the patient was planned for endovascular intervention.

**Figure 3:**
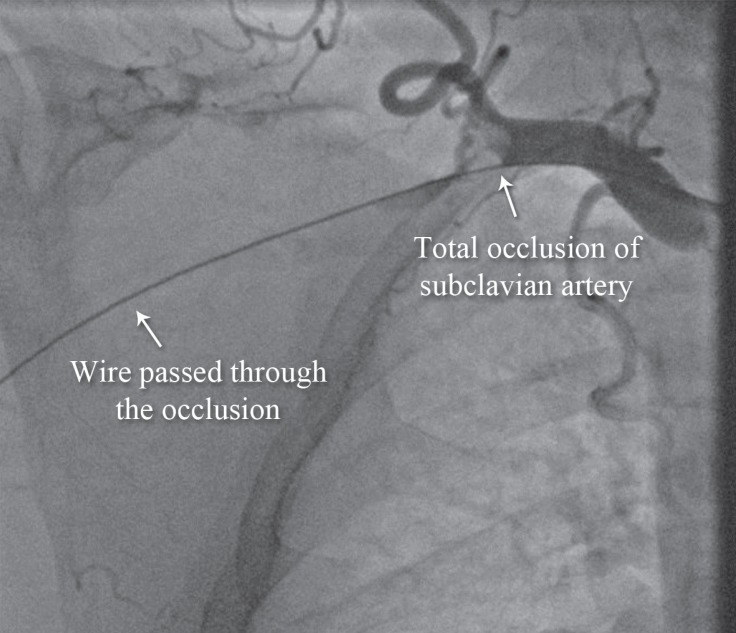
Anteroposterior view of the right upper extremity angiogram shows total occlusion of the right the subclavian artery (Case # 2).

**Figure 4:**
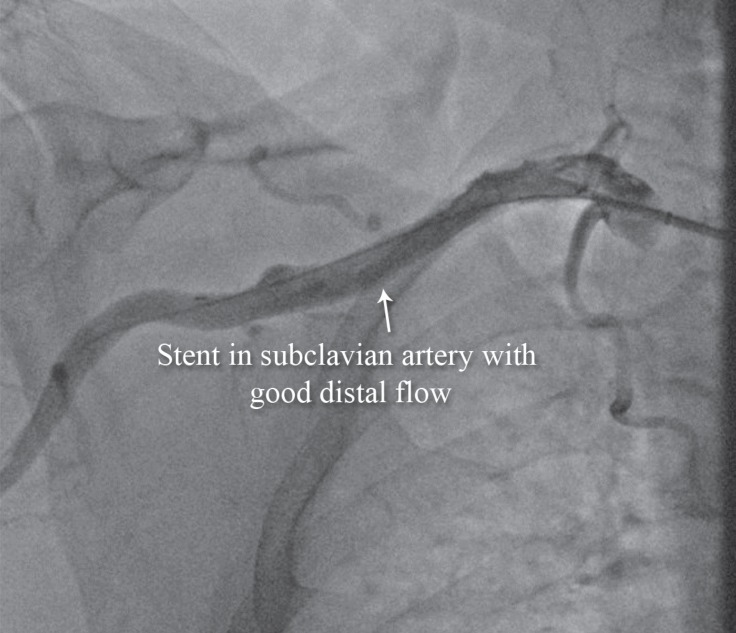
Anteroposterior view of the right upper extremity shows successful angioplasty of the subclavian artery with excellent results (Case # 2).

**Figure 5:**
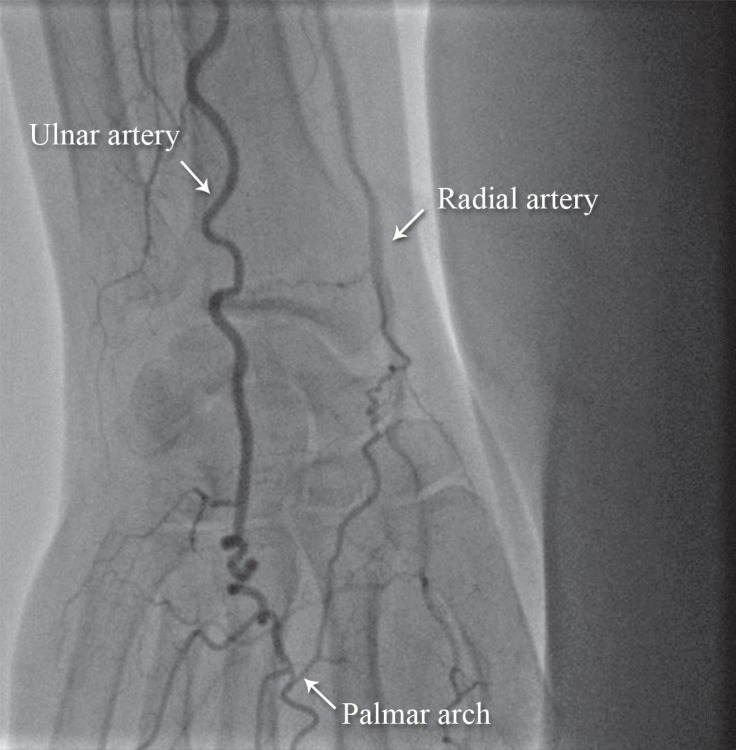
Anteroposterior view of the right upper extremity shows restoration of the palmar arch flow after successful angioplasty (Case # 2).

We passed a stiff 0.035-mm hydrophilic Terumo wire (Figure 3) and then passed a support catheter distally. We did an injection to ensure that we were inside the true lumen. Next, we deployed an 8 × 120 self-expandable stent and postdilated it with an 8 × 60 balloon. The final result was excellent (Figure 4), and the palmar arch had a good distal filling (Figure 5).

***Case #3***

**Figure 6:**
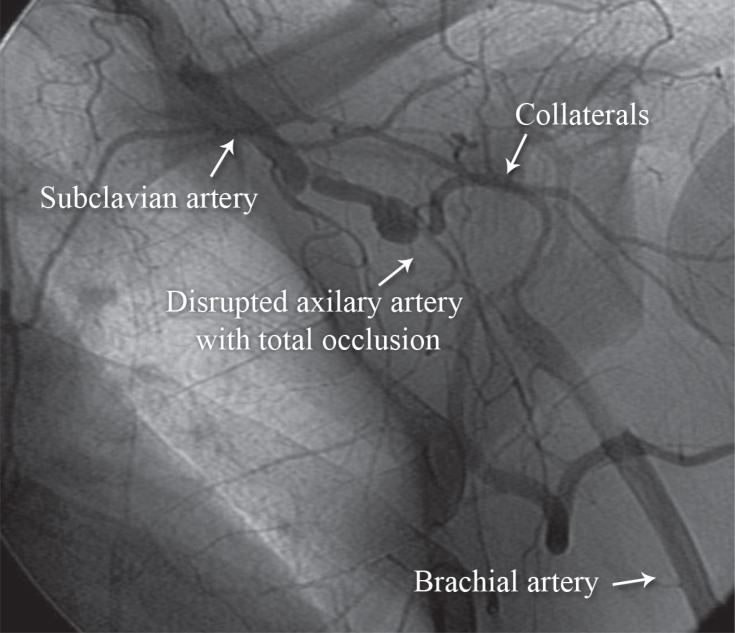
Anteroposterior view of the left upper extremity shows preprocedural total occlusion of the left subclavian artery (Case # 3).

**Figure 7:**
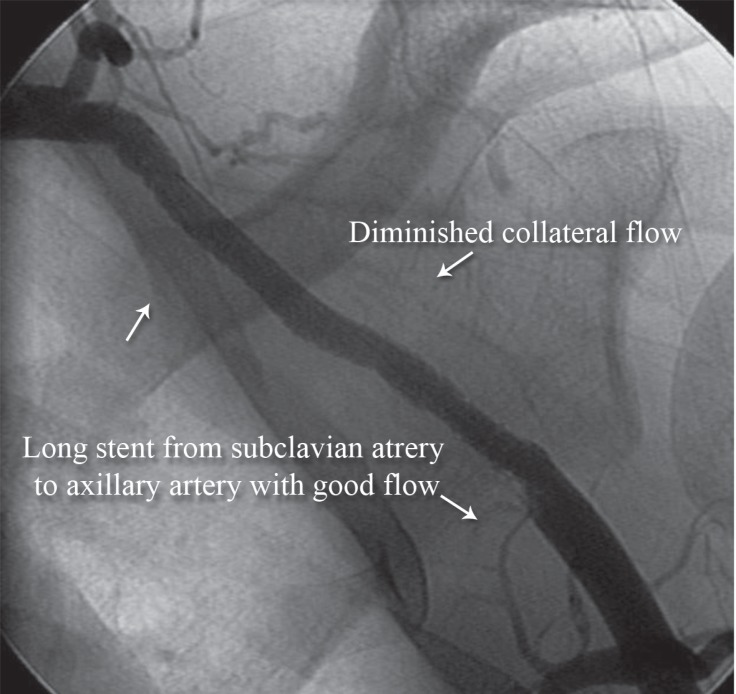
Anteroposterior view of the left upper extremity shows successful revascularization of the left subclavian artery (Case # 3).

A 28-year-old man, a case of motorcycle accident with multiple fractures and the dissection and disruption of the left axillary artery, was rejected by a surgeon due to severe edema, dirty wound, and severely disrupted anatomy. We successfully repaired the dissection and disruption of the left axillary artery via the endovascular approach. The preprocedural and postprocedural images are depicted in Figure 6 and Figure 7, respectively.

***Case #4***

**Figure 8:**
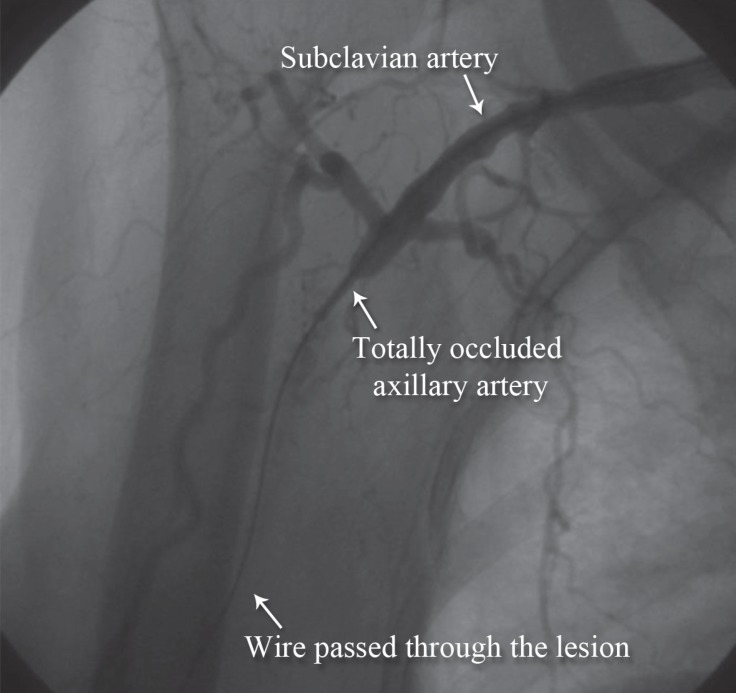
Anteroposterior view of the right upper extremity shows occluded subclavian artery with the passage of the guide wire (Case # 4).

**Figure 9:**
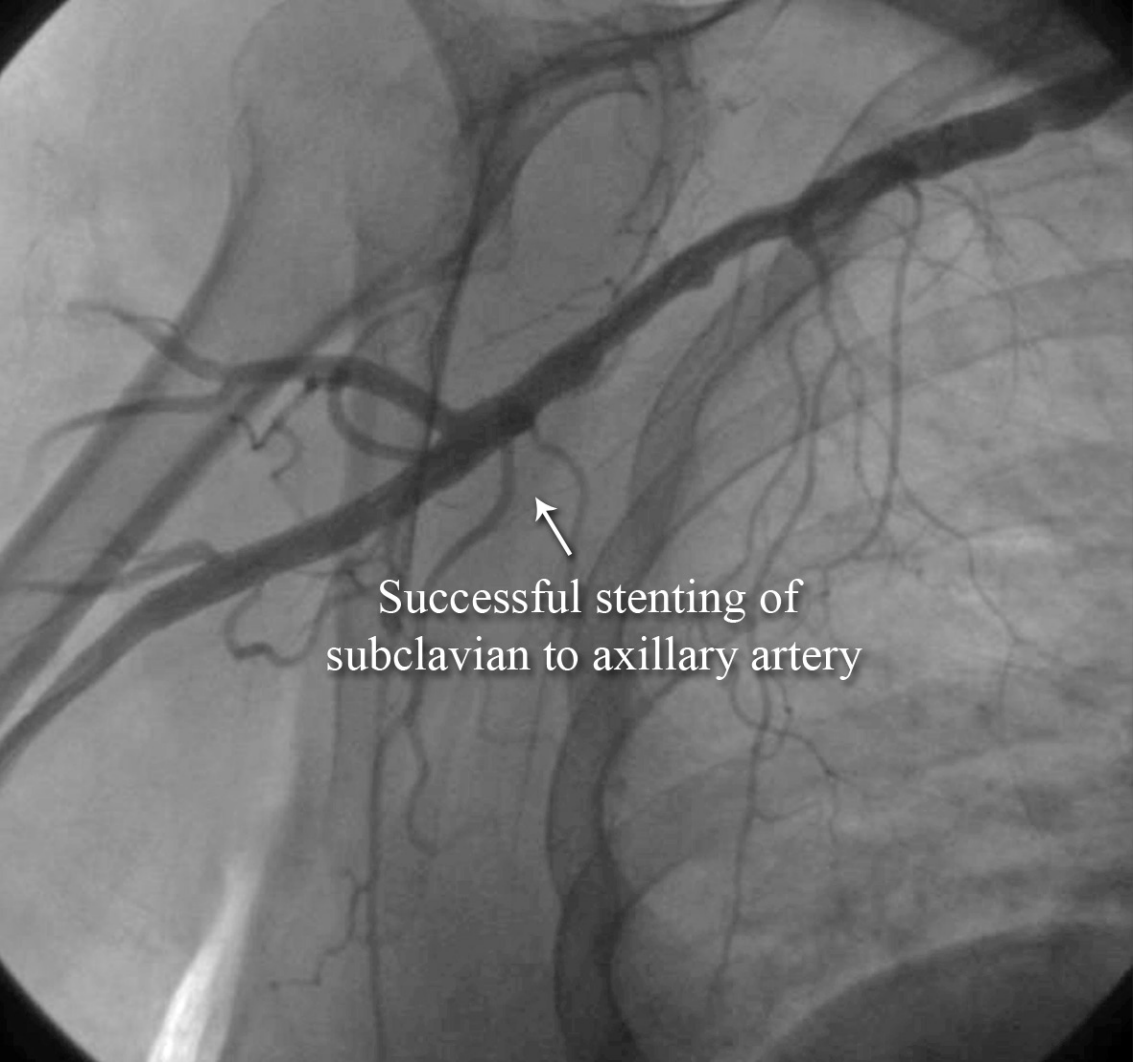
Anteroposterior view of the right upper extremity shows successful stenting of the subclavian artery (AP view, Case # 4).

A 52-year-old woman, a case of fall and blunt trauma to the right shoulder, presented with the numbness and loss of the right upper extremity pulses along with severe pain but no discoloration. After passing a stiff 0.035-mm hydrophilic Terumo wire (Figure 8), we accomplished stenting with an 8 × 40 mm self-expandable stent and postdilated it with a 7 × 30 mm balloon with good results (Figure 9).

## Discussion

Subclavian–axillary injuries are commonly associated with penetrating rather than blunt traumas and consist of dissections, avulsions, and pseudoaneurysms. Surgical repair carries high complication rates, which is why endovascular approaches have gained popularity. The literature contains several reports of successful endovascular repair with covered stent grafts^4^^-^^9^ yielding good short-term^4^ and midterm results.^6^ However, long-term durability has not been established yet. 

We used self-expandable non-covered stents because our selected cases had limb ischemia due to the dissection of the subclavian–axillary artery rather than pseudoaneurysms or avulsions or other bleeding injuries. The flexibility of these stents affords smooth deployment even in tortuous vessels. Additionally, the final length is under the control of the operator depending on the diameter of the expansion of the stent. Moreover, their flexibility augments the function of the flexure areas of the shoulder without limiting motion. We achieved excellent immediate results with limb salvage and without complications. We have had 1 of our patients under follow-up for 4 years; the other 3 have been under follow-up for 1 year now. They all have good peripheral pulses and patent revascularized arteries according to duplex sonography.

We recommended further multicenter studies with larger sample volumes. Long-term follow-ups are needed to evaluate the efficacy of non-covered self-expandable stents in the setting of the traumatic dissection of the subclavian–axillary artery.

## Conclusion

The endovascular repair of the traumatic dissection of the subclavian–axillary artery can be safely done with non-covered self-expandable stents.

## Notes:

***This paper should be cited as: ***Ostovan MA, Kojuri J, Dehghani P. Endovascular Repair of the Traumatic Dissection of the Subclavian-Axillary Artery: Report of Four Cases. J Teh Univ Heart Ctr 2017;12(2):88-91.
